# Antimicrobial resistance crisis: could artificial intelligence be the solution?

**DOI:** 10.1186/s40779-024-00510-1

**Published:** 2024-01-23

**Authors:** Guang-Yu Liu, Dan Yu, Mei-Mei Fan, Xu Zhang, Ze-Yu Jin, Christoph Tang, Xiao-Fen Liu

**Affiliations:** 1 Department of Immunology and Pathogen Biology, School of Basic Medical Sciences, Hangzhou Normal University, Key Laboratory of Aging and Cancer Biology of Zhejiang Province, Key Laboratory of Inflammation and Immunoregulation of Hangzhou, Hangzhou Normal University, Hangzhou, 311121 China; 2 National Key Discipline of Pediatrics Key Laboratory of Major Diseases in Children Ministry of Education, Laboratory of Dermatology, Beijing Pediatric Research Institute, Beijing Children’s Hospital, Capital Medical University, National Center for Children’s Health, Beijing, 100045 China; 3 Robert and Arlene Kogod Center on Aging, Mayo Clinic, Rochester, MN 55905 USA; 4 Department of Biochemistry and Molecular Biology, Mayo Clinic, Rochester, MN 55905 USA; 5 Department of Molecular and Human Genetics, Baylor College of Medicine, Houston, TX 77030 USA; 6 Sir William Dunn School of Pathology, University of Oxford, Oxford, OX1 3RE UK; 7 Institute of Antibiotics, Huashan Hospital, Fudan University, Key Laboratory of Clinical Pharmacology of Antibiotics, National Health Commission of the People’s Republic of China, National Clinical Research Centre for Aging and Medicine, Huashan Hospital, Fudan University, Shanghai, 200040 China

**Keywords:** Antibiotic, Artificial intelligence (AI), Clinical development, Machine learning (ML), Antimicrobial peptide, Phage therapy, Antibiotic stewardship

## Abstract

Antimicrobial resistance is a global public health threat, and the World Health Organization (WHO) has announced a priority list of the most threatening pathogens against which novel antibiotics need to be developed. The discovery and introduction of novel antibiotics are time-consuming and expensive. According to WHO’s report of antibacterial agents in clinical development, only 18 novel antibiotics have been approved since 2014. Therefore, novel antibiotics are critically needed. Artificial intelligence (AI) has been rapidly applied to drug development since its recent technical breakthrough and has dramatically improved the efficiency of the discovery of novel antibiotics. Here, we first summarized recently marketed novel antibiotics, and antibiotic candidates in clinical development. In addition, we systematically reviewed the involvement of AI in antibacterial drug development and utilization, including small molecules, antimicrobial peptides, phage therapy, essential oils, as well as resistance mechanism prediction, and antibiotic stewardship.

## Background

Antimicrobial resistance (AMR) is a natural phenomenon wherein microorganisms, including bacteria, viruses, fungi, and parasites, develop the ability to survive the drugs designed to kill them. The misuse and overuse of antibiotics in human medicine, animal agriculture, and the environment have accelerated the emergence and spread of AMR. This phenomenon renders once-effective treatments ineffective, leading to prolonged illnesses, increased mortality rates, and higher healthcare costs. Thus, AMR is a serious and foremost global threat to human health that requires practical actions urgently. The Global Antimicrobial Resistance and Use Surveillance System launched by the World Health Organization (WHO) revealed that AMR is on the rise and already a leading cause of death [1, 2]. Globally, it was estimated that, in 2019 alone, approximately 4.95 million deaths were linked to bacterial AMR, with 1.27 million deaths specifically attributed to bacterial AMR [1]. The highest all-age death rate due to resistance was observed in Western sub-Saharan Africa, with 27.3 deaths per 100,000 individuals (20.9–35.3) [1]. According to the data from the Centers for Disease Control and Prevention report, AMR to at least first-line antibiotics accounts for more than two million infections in the US alone each year and at least 23,000 deaths [3]. There were more than 2.8 million infections caused by antibiotic-resistant bacteria in the US in 2019 [4]. It is estimated that AMR will cause 10 million deaths each year by 2050 [5]. The Infectious Disease Society of America has highlighted 6 pathogens including *Enterococcus faecium*, *Staphylococcus aureus* (*S*. *aureus*), *Klebsiella pneumoniae* (*K*. *pneumoniae*), *Acinetobacter baumannii* (*A*. *baumannii*), *Pseudomonas aeruginosa* (*P*. *aeruginosa*) and *Enterobacter* spp. as “ESKAPE” organisms, which pose the highest threat to human lives, owing to their fast-growing antibiotic resistance [6]. The WHO has published an antibiotic-resistant “priority pathogen” list to help drug developers target the pathogens that urgently need novel antibiotics. AMR has also become a public health concern in China. According to data from the Chinese Antimicrobial Surveillance Network, the resistance rate of carbapenem-resistant Gram-negative bacteria has shown a significant increase. Notably, carbapenem-resistant *A*. *baumannii* has risen from 39.0 to 71.9%, while carbapenem-resistant *K. pneumonia* has surged from 2.9 to 24.2% from 2005 to 2022 [7]. Additionally, methicillin-resistant *S*. *aureus* has been consistently detected at a high rate of approximately 30% in recent years (www.chinets.com) [7].

The number of novel antibiotics developed and approved has gradually decreased over the past decade, with only 4 novel antibiotics approved between 2010 and 2014 [8], resulting in limited treatment options for antibiotic-resistant bacterial infections in clinics. Historically, antibiotics were mostly discovered by screening secondary metabolites with antibacterial activities from soil microbes [9]. Unfortunately, the discovery of novel antibiotics is becoming increasingly difficult due to the rediscovery problem where identical compounds were isolated repeatedly [10]. Thus, new drug development is insufficient to meet the demands of clinical treatment, especially for those pathogens on the WHO priority list. Artificial intelligence (AI), a field of computer science, refers to the development of intelligent machines capable of executing tasks typically requiring human-like intelligence in an objective fashion [11]. AI technologies present innovative approaches and have increasingly integrated into a wide range of disciplines to accelerate scientific discoveries, especially in medicine where AI has empowered the discovery of novel drugs and expedited the overall drug development and clinical research process [12–14]. Without exceptions, AI has constituted a central part of concerted interdisciplinary efforts to tackle the crisis of AMR [15]. In this review, we will discuss the progress and challenges of antibacterial drugs in clinical and preclinical development, as well as novel AI-based methodologies in antibacterial drug development, with a particular focus on new drug design, structure optimization, and exploration of new mechanisms of action (MOA).

## Antibacterial agents in clinical development

**Fig. 1:**
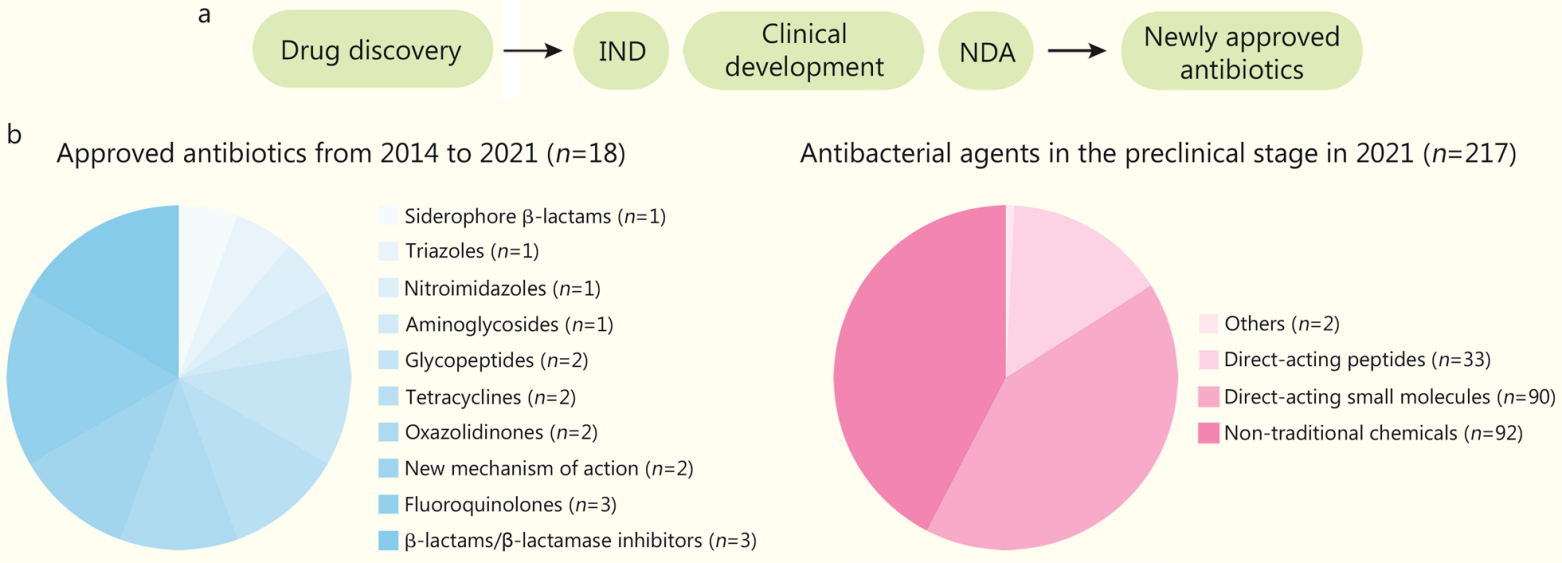
The pipeline (**a**) and the status (**b**) of novel antibiotic development. Novel drugs discovered in laboratories need to go through several stages including Investigational New Drug (IND) application, clinical development, and New Drug Application (NDA) before they become approved. Non-traditional chemicals include bacteriophage/phage products (*n* = 28), indirect-acting small molecules (*n* = 23), large molecules (*n* = 19), biologics (antibody or others, *n* = 8), immunomodulators (*n* = 7), nucleic acid-based products (*n* = 4), indirect-acting peptide (*n* = 2), and microbiome modifying agents (*n* = 1)

The development of new drugs is a time-consuming and resource-intensive process that involves synthesizing thousands of chemicals derived from existing drugs or mechanisms. This is followed by preliminary activity and toxicity screening to identify one or two potential candidates. The existing development of new antibacterial treatments is far from adequate to address the rapid increase of antibiotic resistance, according to the WHO’s annual report on the pipeline of drugs. From 2014 to the end of 2021, 18 antibiotics, including one for the treatment of extensively drug-resistant tuberculosis, have been approved and available (Table 1). Among these antibiotics, 16 were approved by the US Food and Drug Administration (FDA), 12 were approved by the European Medicines Agency, 1 was approved by the Central Drugs Standard Control Organization of the Government of India, 1 (contezolid) by Chinese National Medical Products Administration, and 1 by Pharmaceuticals and Medical Devices Agency (Japan). Moreover, only vaborbactam and lefamulin have new MOA. The rest of the antibiotics belong to known classes, including fluoroquinolones (3/16), β-lactam/β-lactamase inhibitors (3/16), glycopeptides (2/16), tetracyclines (2/16), oxazolidinones (2/16), aminoglycosides (1/16), nitroimidazoles (1/16), triazoles (1/16), and siderophore β-lactams (1/16) (Fig. 1).

**Table 1:** Approved antibiotics from 2014 to 2021

Drug	Target pathogen	Class	Approval year	Approved by
Dalbavancin	Gram-positive pathogens	Glycopeptide	2014	US FDA; EMA
Tedizolid	Gram-positive pathogens	Oxazolidinone	2014	US FDA; EMA
Oritavancin	Gram-positive pathogens	Glycopeptide	2014	US FDA
Ceftolozane/tazobactam	β-lactamase enzyme producing bacteria	β-lactam/β-lactamase inhibitor	2014	US FDA; EMA
Cefazidime/avibactam	CRE	β-lactam/β-lactamase inhibitor	2015	US FDA; EMA
Isavuconazonium	Antifungal	Triazole	2015	US FDA
Delafloxacin	Gram-positive pathogens	Fluoroquinolone	2017	US FDA; EMA
Vaborbactam/meropenem	CRE	β-lactam/β-lactamase inhibitor	2017	US FDA; EMA
Plazomicin	CRE	Aminoglycoside	2018	US FDA
Eravacycline	CRE	Tetracycline	2018	US FDA; EMA
Omadacycline	MRSA and CRE	Tetracycline	2018	US FDA
Relebactam + imipenem/Cilastatinilastatin	CRE, and potential activity for CRPA	β-lactam/β-lactamase inhibitor	2019	US FDA; EMA
Lefamulin	MSSA	Pleuromutilin	2019	US FDA; EMA
Pretomanid	XDR-TB	Nitroimidazole	2019	US FDA; EMA
Lascufloxacin	Gram-positive pathogens	Fluoroquinolone	2019	PMDA
Cefiderocol	CRAB, CRPA, CRE	Siderophore β-lactam (cephalosporin)	2019	US FDA; EMA
Levonadifloxacin	Gram-positive pathogens	Fluoroquinolone	2020	CDSCO
Contezolid	MRSA	Oxazolidinone	2021	US FDA; EMA; China

*CRE* carbapenem-resistant *Enterobacteriaceae*, *MRSA* methicillin-resistant *Staphylococcus aureus*, *CRPA* carbapenem-resistant *Pseudomonas aeruginosa*, *MSSA* methicillin-susceptible *Staphylococcus aureus*, *XDR-TB* extensively drug-resistant tuberculosis, *CRAB* carbapenem-resistant *Acinetobacter baumannii*, *FDA* Food and Drug Administration, *EMA* European Medicines Agency, *PMDA* Pharmaceuticals and Medical Devices Agency, *CDSCO* Central Drugs Standard Control Organization

According to WHO’s reports, there was a total of 42 new therapeutic agents in 2017 (including 33 antibiotics targeting bacterial priority pathogens and 9 biologicals), and 59 in 2021 (27 antibiotics and 32 biologicals) [16, 17], indicating a rapid increase of biologicals (including monoclonal antibody, phage endolysin, polyclonal antibody, etc.) development over traditional antibiotics in recent years. These biologicals were developed in response to the growing demand for novel antibacterial agents with new targets and MOA. These biologicals have promoted a shift of developers’ focus from targeting narrow-spectrum agents to a single pathogen specifically. However, there are only 6 of the 27 antibiotics (including New Drug Application, and I–III Phase clinical trials) considered to be innovative drugs according to WHO criteria [17]. Although almost half of them are still β-lactam/β-lactamase inhibitor combinations, they were markedly safer and more promising combinations for treating infections caused by Gram-negative bacteria [18]. As for extensively drug-resistant Gram-negative bacterial infections, only tigecycline (2005 approved by the FDA and available in China since 2010), polymyxins (which re-entered the Chinese market in 2017), and ceftazidime/avibactam (2019) are available clinically.

### Polymyxin derivatives in clinical development

Polymyxins, which belong to lipopeptides, are considered as last-line antibiotics and have raised great interest from scientists and pharmaceutical companies to optimize their efficacy and toxicity. Polymyxins are cyclic lipopeptides with 5 positively charged 2,4-diamino butyric acid groups [19]. They are an old class of antibiotics developed in the 1950s and were replaced by other safer antibiotics due to their renal toxicity [20]. However, the emergence of extensively-resistant Gram-negative bacteria, which are resistant to multiple classes of antibiotics and only susceptible to one or two antibiotics, has posed significant challenges to clinics. Polymyxins, with a susceptible rate as high as 98% against these extensively-resistant Gram-negative bacteria, were re-introduced into clinical practice and are now regarded as a last-line of defense against carbapenem-resistant Gram-negative bacteria [21, 22]. There are two polymyxins used in clinics: the prodrug colistimethate (the active form is colistin or polymyxin E) and polymyxin B sulfate. The structures of components of polymyxin E and polymyxin B are similar, and they possess similar antibacterial activity [21]. Polymyxins can interact with the negatively charged lipid A of lipopolysaccharide and lead to the disruption of the cell membrane and, ultimately, cell death [23]. Nevertheless, dose-limiting nephrotoxicity (due to the reabsorption in renal tubular cells) and their narrow therapeutic window are key factors hampering the use of polymyxins in clinical settings [24–27]. Furthermore, the poor tissue distribution of polymyxins in the lung after intravenous administration results in low efficacy in pulmonary infections [28–30]. Recent drug development therefore has focused on optimizing the structure of polymyxins, with the hope to obtain drug candidates with better efficacy profiles and less renal toxicity. Of note, several derivatives of polymyxins are in clinical development, specifically targeting carbapenem-resistant *A. baumannii*, *P. aeruginosa* and *K*. *pneumoniae* and are expected to have an improved therapeutic window [31–33].

Based on the structure–activity relationships (SAR), polymyxin derivatives were synthesized and screened for their antibacterial activity, cytotoxicity and renal toxicity. SPR206, a novel polymyxin derivative with an amine-containing N-terminal moiety, was shown to have high antimicrobial activity against carbapenem-resistant Gram-negative bacteria and lower kidney cell cytotoxicity (11.6-fold less cytotoxic in HK-2 kidney cells than polymyxin B) [31]. Furthermore, it exhibits reduced in vivo nephrotoxicity, as indicated by biomarker levels (including KIM-1, cystatin-C, and albumin in urine) after a 25 mg/kg dose regimen for SPR206, similar to those for polymyxin B at 12.5 mg/kg dose [34]. SPR206 showed lower minimum inhibitory concentrations (MICs) (0.12 to 0.50 mg/L) than polymyxins against *A. baumannii*, *K. pneumoniae*, and *P. aeruginosa* [34, 35]. The drug has now completed first-in-human studies, which evaluated its safety, tolerability, and pharmacokinetics in healthy subjects [31]. Single dose escalation from 10 to 400 mg, multiple dosing (every 8 h for 7 d), and multiple doses of 100 mg (every 8 h for 14 d) were safe and well tolerated in healthy subjects [31]. SPR206 demonstrated a urinary recovery of up to 50% in critically ill patients [31], whereas polymyxin B only showed a urinary recovery of 4% in critically ill patients [36] and 4–8% in healthy subjects [37]. The higher urinary recovery highlights improved safety and hence the potential clinical application of urinary tract infections (UTIs) for SPR206.

According to the interactions between polymyxins and lipid A, a SAR-based mechanistic model was built to evaluate and design new structures with potent antimicrobial activity [38]. By applying this model, combining with the structure–toxicity relationship (STR) and the structure-pharmacokinetic relationship (SPR), a synthetic lipopeptide F365 (QPX9003) was developed [32]. Benefitting from the optimization of non-conserved positions in the polymyxin scaffold, QPX9003 is considered a success in uncoupling therapeutic efficacy from toxicity. Compared to available polymyxins, this compound has a wider therapeutic window, with up to a fourfold increase in the maximal tolerable dose relative to polymyxin B and colistin in an acute toxicity mouse model. Additionally, it demonstrated reduced nephrotoxicity, as there was no sign of nephrotoxicity even at a dose up to 72 mg/(kg·d) in mouse model [32]. It also displayed distinct pharmacological characteristics, with more than threefold free drug exposure and at least fourfold urinary recovery compared to polymyxin B [32]. Furthermore, it showed improved drug exposure in the lung with eightfold lower lung surfactant binding to achieve efficacious drug exposure. Currently, the compound has entered Phase I clinical trials [32].

MRX-8, a novel polymyxin analog, possesses a fatty acyl tail attached to its polymyxin B scaffold via an ester bond [39]. The ester bond can be cleaved to yield tail-less and less toxic metabolites [40]. MRX-8 was designed to treat multidrug-resistant Gram-negative bacteria and alleviate renal toxicity by breaking its ester bond [33]. It was developed by applying a “soft drug design” approach, which aims to design less toxic drugs with a wider therapeutic window [41]. In vitro studies identified the MIC_50_ and MIC_90_ values of MRX-8 against Gram-negative isolates collected from 2017–2020 in the United States (1314 clinical isolates from the SENTRY Antimicrobial Surveillance Program [42]), which were 0.12 and 0.25 mg/L, respectively, against *Enterobacterales*; and 0.5 and 1.0 mg/L, respectively, against *A. baumannii* and *P. aeruginosa* [43]. Among 765 clinical isolates randomly collected from 2017 to 2020 in China, the MIC_50_/MIC_90_ of MRX-8 was 0.060/0.125 mg/L, respectively, for carbapenem-resistant *Escherichia coli* (*E. coli*) isolates; 0.125/0.500 mg/L, respectively, for carbapenem-resistant *K. pneumoniae* isolates; 1/1 mg/L, respectively, for carbapenem resistant *P. aeruginosa* [44]. The lower MIC values of MRX-8 demonstrated its effectiveness against clinically isolated Gram-negative bacteria, including carbapenem-resistant *E. coli*, *K. pneumoniae*, *P. aeruginosa*, and *A. baumannii.* This underscores its potential as valuable therapeutics. More importantly, in neutropenic mouse thigh and lung models, MRX-8 demonstrated potent activity against *P. aeruginosa*, *A. baumannii*, *K. pneumoniae*, and *E. coli* infection before entering a Phase I clinical trial [33, 43].

### Antimicrobial peptides (AMPs) in clinical development

New antimicrobial biologicals mainly include antibodies, bacteriophages, phage-derived enzymes, and AMPs. AMPs, usually composed of 2–50 amino acids, are produced by multicellular organisms as a defense mechanism against pathogenic microbes [45]. AMPs exert antimicrobial activities through direct interactions with bacterial membranes, which lead to membrane perturbation and disruption of membrane-associated physiological events such as cell wall synthesis, cell division, and translocation across the membrane [46]. A growing body of research is focused on developing AMPs in addressing AMR for several reasons. Firstly, during infections, bacteria frequently reside in biofilms, which are extracellular polymeric matrices that display high resistance to antibiotics [47]. AMPs could outperform traditional antibiotics in this regard as they possess anti-biofilm activities [48]. Secondly, AMPs also act as important effectors and regulators of the innate immune system. They can enhance phagocytosis, wound healing, and angiogenesis, as well as have adjuvant activity in promoting the development of adaptive immunity against an invading pathogen [49, 50].

Examples of important peptides that exhibit potent antimicrobial activities include β-hairpin peptides [51], polyphemusin I [52], and IB-367 (iseganan, which failed in Phase III clinical trials for indications of oral mucositis) [53]. These AMPs have either been discovered, modified, or designed using AI, and they serve as exemplars of the potential of applying AI in the design of novel peptides against drug-resistant bacteria. Some examples of peptides have entered clinical development and the Investigational New Drug Application phase. According to the 2021 WHO report, out of 217 chemicals investigated, 15.2% are direct-acting peptides (*n* = 33), compared with direct-acting small molecules (41.5%, *n* = 90) and other non-traditional chemicals (42.4%, *n* = 92) [16] (Fig. 1). The report demonstrated an increasing prevalence of biologicals in both clinical and preclinical product pipelines. Moreover, the preclinical agents predominantly targeted *P. aeruginosa* or *S. aureus,* showing a clear shift from broad-spectrum antibiotics to narrow-spectrum agents focusing on a single pathogen [16].

Reltecimod (AB103) is a synthetic octapeptide, which is a mimetic of the second CD28 dimer interface domain [54]. CD28 is an antigen expressed on CD4^+^ and CD8^+^ T cells, which can enhance the release of cytokines, including interleukin (IL)-2 and IL-4 [55]. Reltecimod blocks the binding of superantigens from Gram-positive pathogens to CD28 and impairs endotoxin-mediated activation of T cells in Gram-negative bacterial infections [56]. AB103 was demonstrated to reduce mortality in mouse models of polymicrobial and Gram-negative bacterial infections [54, 57]. A clinical trial has also shown the good safety profiles of a single intravenous dose of AB103 at 0.25 or 0.50 mg/kg [58]. Moreover, a Phase III clinical trial has been completed and demonstrated that early treatment of reltecimod in severe necrotizing soft tissue infections resulted in a significant improvement in the primary necrotizing infection according to the clinical composite endpoint [59]. The primary endpoint, which used Necrotizing Infection Clinical Composite Endpoint that required patients to meet all components of the composite score, was 54.3% vs. 40.3% for reltecimod and placebo, and was associated with improved resolution of organ dysfunction (70.9% vs. 53.4% for reltecimod and placebo) and hospital discharge [59].

Murepavadin, an antimicrobial peptidomimetic developed by Polyphor Ltd., binds to the lipopolysaccharide transport protein D (LptD), inhibiting the transportation and location of lipopolysaccharide and disrupting the cell membrane integrity, leading to cell death [60, 61]. It was initially designed and synthesized based on the membranolytic host-defense peptide protegrin I, after which iterative cycles of peptidomimetic library synthesis and screening were performed to improve its antimicrobial activity [61]. It belongs to the class of outer membrane protein targeting antibiotics, which are fully synthetic compounds [62], and exhibit a specific and potent activity against *P. aeruginosa* in vitro and in biofilms [63, 64]. A pharmacokinetics and safety study showed that murepavadin was well tolerated in individuals with a range of renal function (from normal, mild to severe renal function impairment); dose adjustment was warranted in renal dysfunction patients [65]. In a completed Phase II clinical trial, 12 patients with *P. aeruginosa* ventilator-associated pneumonia infections received murepavadin treatment, and 10 (83%) of these patients achieved a clinical cure. The 28-day all-cause mortality rate was 8%, which was far below the 20–40% expected mortality rate [60]. However, the Phase III clinical trials for treating nosocomial pneumonia or ventilator-associated pneumonia caused by *P. aeruginosa* have been terminated due to higher-than-expected kidney injury incidences.

## AI in antimicrobial development

As the scale of biological big data continues to increase, a variety of AI methods for analyzing biological big data have emerged [66]. AI technology can enable computers to learn and improve automatically without explicit programming, and construct and predict models using data. The methodology domain involved in AI mainly includes reasoning, knowledge representation, search for solutions and machine learning (ML) [67]. Deep learning (DL), a constituent of ML, involves a neural network that mimics the structure of the brain and is used to recognize and differentiate patterns of language, images, videos, and various biological data types [68, 69]. DL-related algorithms have advanced rapidly in recent years, and several typical algorithms, including convolutional neural networks, recurrent neural networks, deep reinforcement learning, and particularly, generative adversarial networks, as an unsupervised learning algorithm [70], have been extensively applied in various fields of drug discovery [71–73].

**Fig. 2:**
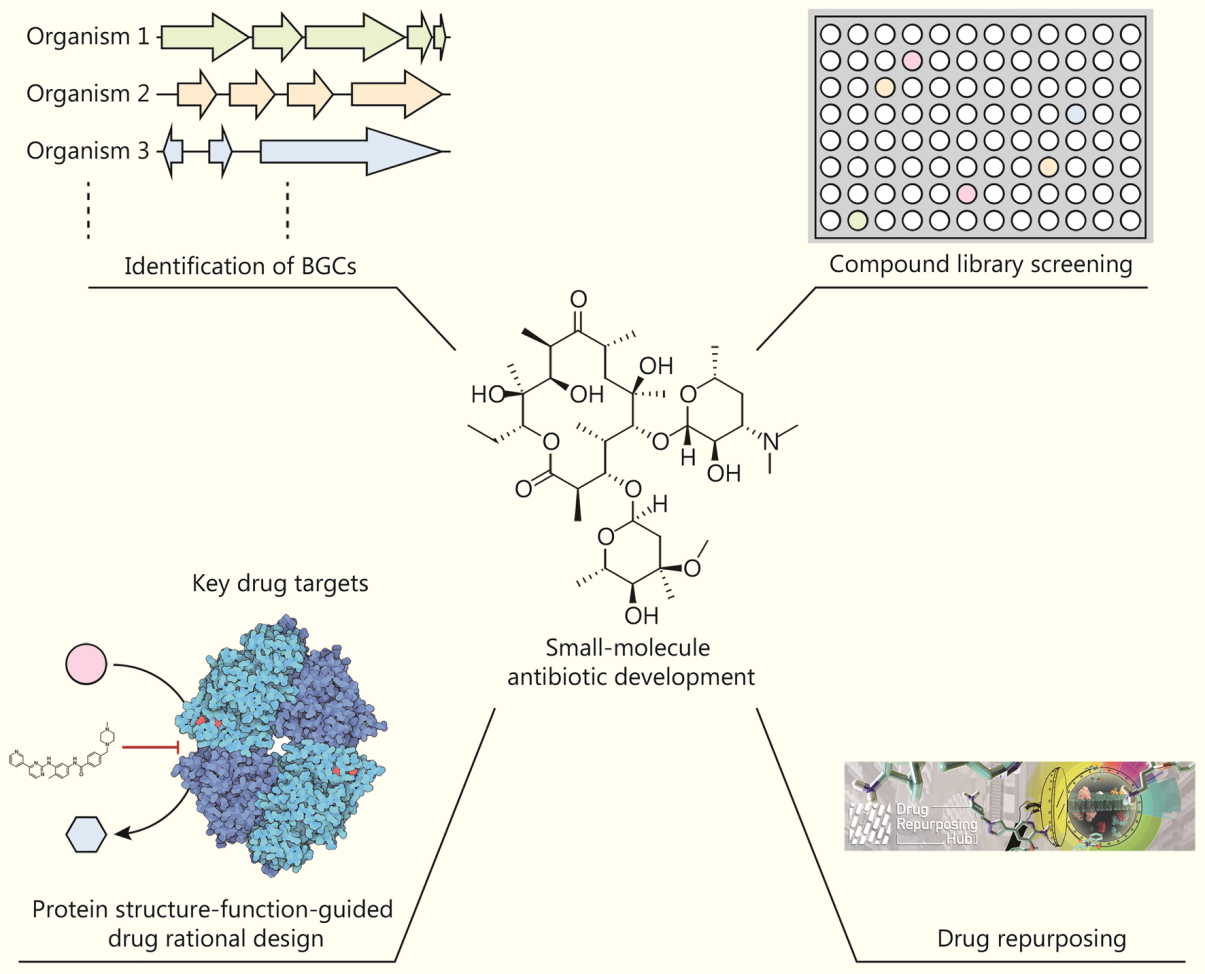
Artificial intelligence (AI) in small-molecule antibiotic development. AI-based methods empower novel small-molecule antibiotic discovery from multiple dimensions, including mining secondary metabolites encoded by biosynthetic gene clusters (BGCs), screening existing compound libraries, and repurposing the Food and Drug Administration (FDA)-approved drugs. AI-based prediction of protein structures and functions, such as AlphaFold2 and RoseTTAFold, remarkably expands the protein space for docking simulations and drug rational design

AI technology constitutes a powerful tool to combat AMR [74–76]. For example, data-driven methods can be used to predict novel antibiotic compounds, while image-based methods can help identify resistant bacteria [77]. AI-assisted compound library screening or new compound structure design can help quickly identify more promising antimicrobial compounds. In addition, AI can leverage known data, such as genomic data, to predict potential resistance sites and related enzymatic functions, laying the groundwork for designing better antibiotics [78]. Furthermore, AI has facilitated target identification and dynamic modeling, peptide design and synthesis, evaluation of SAR and STR, and drug repurposing [79] (Fig. 2). In the following sections, we will categorize antimicrobials into 4 major groups: small molecules, AMPs, phage therapy, and essential oils (EOs). We will also discuss the role of AI in the development of each category.

### AI in small-molecule antibiotic development

The screening of soil microbe-derived secondary metabolites exhibiting antibacterial properties was once the predominant method for identifying novel antibiotics, especially between the 1950s and 1960s, a period known as the golden era of antibiotic discovery [80]. Unfortunately, this approach’s success was impeded by the rediscovery problem, and in later periods, a substantial majority of newly developed antibiotics achieving clinical utilization were classified as analogs of existing antibiotic classes. The enduring efficacy of these analogs faced challenges due to the pervasive prevalence of existing resistance determinants [9]. Ideally, new antibiotic discovery should aim to pursue novel chemotypes with MOAs distinctly dissimilar from existing antibiotics. Such compounds are more likely to evade existing resistance determinants and therefore exhibit prolonged utility over time. Fortunately, AI technologies have been speeding up this process from multiple angles. In this section, we will discuss AI-facilitated small-molecule antibiotic discovery from 4 aspects: identification of biosynthetic gene clusters (BGCs), compound library screening, protein structure–function-guided drug rational design, and drug repurposing (Fig. 2).

#### Small molecule antibiotic development by BGCs

BGCs serve as natural reservoirs encoding a plethora of secondary metabolites with the potential to become novel antibacterials [81]. However, tapping into the full potential of BGCs is hampered by the fact that many BGCs belong to organisms that cannot be cultured under laboratory conditions. Even for those that are culturable, BGCs could be cryptic or remain transcriptionally silent. Therefore, there is a strong demand for in silico prediction of BGCs through genome mining. Despite traditional rule-based systems such as antibiotics and secondary metabolite analysis shell (antiSMASH) [82] and Antibiotic Resistant Target Seeker (ARTS) [83] having already shown great success, AI has been playing an increasingly important role [81]. DeepBGC utilized a DL algorithm to uncover novel BGC classes and predict products’ chemical activity [84]. Another DL-based method, DeepRiPP, integrated both genomic and metabolomic data for predicting ribosomally synthesized and posttranslationally modified peptides [85]. PRISM 4, on the other hand, achieved predictions using the sequence information of the BGC to predict the chemical structures of the secondary metabolites, from which the activities can be inferred [86]. Walker et al. [87] also accomplished highly accurate activity prediction by combining BGCs and profiles of resistance markers.

#### MOA driven drug screening

Conventional drug screening is limited by the size of the compound library and is often unable to determine the MOA of the hits. To overcome these limitations, Johnson et al. [88] developed a novel paradigm termed PROSPECT (primary screening of strains to prioritize expanded chemistry and targets) by performing a primary chemical screen on *Mycobacterium tuberculosis* (*M. tuberculosis*) hypomorphs (mutant strains depleted in essential targets). As a result, over 8.5 million chemical-genetic interaction profiles were generated, and with the help of supervised learning, a significantly increased number of inhibitors against essential targets such as DNA gyrase and folate metabolism were identified [88]. Moreover, ML-based screens enable the exploration of extensive chemical spaces in silico. This, in turn, substantially enhances the probability of unearthing structurally and functionally novel compounds imbued with potent antibacterial properties. Taking *A. baumannii*, a nosocomial pathogen notorious for drug resistance, as an example, Liu et al. [89] trained a message-passing deep neural network with growth inhibition data from around 7500 FDA-approved compounds, which then discovered aubacin, a narrow-spectrum antibiotic with a novel MOA targeting lipoprotein trafficking.

#### AI in protein structure prediction and drug design

In addition to mining for novel secondary metabolites and screening existing compound libraries, AI-based approaches can also promote the rational design and optimization of drugs. The basis of rational design lies in understanding the three-dimensional structure, and in many cases, protein–protein interactions of the drug target. Although experimental determination remains the gold standard for protein structures, the process is low-throughput, and for many proteins, it remains technically challenging. Fortunately, this was revolutionized by the advent of the neural network AlphaFold2 by DeepMind, which achieved structure prediction for the majority of proteins with near-experimental accuracy [90, 91]. Inspired by the principles of AlphaFold2, Baek et al. [92] created RoseTTAFold, a three-track neural network for structure prediction that achieved accuracy approaching that of AlphaFold2. RoseTTAFold also enables the prediction of protein–protein interactions directly from protein sequences, avoiding the need for subunit structure prediction and docking [92].

To apply these powerful tools for antibiotic discovery, Wong et al. [93] conducted molecular docking simulations using structures of the *E. coli* essential proteome predicted by AlphaFold2, along with hundreds of active/inactive antibacterials, and performed in vitro enzymatic assays to assess the model performance. Unfortunately, the model showed, on average, weak performance across 12 essential targets with an area under the receiver characteristic curve (AUROC) of 0.48, and this was attributed to the docking method rather than AlphaFold2 predictions [93]. To improve performance, a combination of ML-based scoring functions was employed to refine the docking calculations, leading to a significantly increased AUROC [93]. Overall, this indicates that there are still significant roadblocks to accurately predicting how drug candidates bind to their targets, even with the help of AlphaFold2, and improving ligand binding poses predicted by docking simulations to AlphaFold2 models holds the key [93, 94].

Both AlphaFold2 and RoseTTAFold use multiple-sequence alignments as inputs for DL, sacrificing time for database searching for prediction accuracy. To overcome this limitation, Fang et al. [95] developed HelixFold-Single, an algorithm that combines a protein language model and geometric learning from AlphaFold2. Due to the avoidance of multiple-sequence alignment, compared to AlphaFold2, HelixFold-Single reduces prediction time by 99.9% for proteins smaller than 100 amino acids and still by 96% for proteins larger than 800 amino acids, greatly facilitating applications such as high-throughput protein structure predictions [95].

With the success of AlphaFold2 and RoseTTAFold, the next significant question pertains to predicting the function of proteins directly from their primary sequence [96]. Answering this question could lead to a substantial expansion of potential targets for antibiotic discovery. Conventional alignment-based methods struggle to detect remote homology, and approximately one-third of bacterial proteins remain unannotated [97]. To address this gap, Gligorijević et al. [98] developed DeepFRI, a graph convolutional model consisting of a two-stage architecture that incorporates both protein structure and sequence represented in a long short-term memory language model. This allows DeepFRI to extract local sequence and global structure features, bypassing limitations imposed by homology-based function transfer. In addition, Bileschi et al. trained neural networks ProtCNN and ProtENN using curated seed sequences from Pfam [99, 100]. These models outperformed existing methods such as TPHHM and BLASTp [100]. Moreover, when combined with existing methods, DL models can learn complementary information and offer approximately 6.8 million new sequence region annotations, significantly expanding the coverage of Pfam by more than 9.5% [100].

Structural homology is more conserved and, hence, of greater value in predicting protein functions than sequence homology across long evolutionary distances. Based on this principle, Hamamsy et al. [101] developed a workflow consisting of two consecutive DL methods, TM-Vec and DeepBLAST. TM-Vec employs TM-scores as a metric to search for structural similarities in large sequence databases directly from sequence pairs without intermediate structural prediction. DeepBLAST then performs subsequent structural alignments. This workflow outperforms existing sequence-alignment methods for remote homology detection and ultimately expedites protein function prediction [101].

The concept of rational design goes beyond predicting protein structures and functions. Inspired by natural language processing (NLP), Mansbach et al. [102] took fragment-based representations and drug activity to train an ML algorithm termed “Hunting FOX”. Unlike conventional fragment-based drug design approaches that rely on predefined fragments, Hunting FOX enables the extraction of all potential fragments from compounds to form hybrids with novel functionalities [102]. With Hunting FOX, Mansbach et al. [102] identified a chemical vocabulary related to high permeation into *P. aeruginosa* and validated this approach in vitro.

There are also novel approaches to drug design and screening that involve entirely new structures and operate at a much faster pace than ever before. De novo drug design, a computational approach, designs new chemical structures from atomic building blocks without being restricted by SAR or drug-target interaction relationships. Deep reinforcement learning, which combines artificial neural networks (ANNs) with reinforcement-learning architectures, has been successfully employed for de novo drug design [103]. Monte Carlo tree search integrated with symbolic AI was applied to guide the selection of promising retrosynthetic steps and search for drug candidates. This approach was 30 times faster than the traditional computer-aided search method [104].

#### AI in drug repurposing

Drug repurposing, involving the identification of new applications for existing drugs, has been gaining momentum in both the public and private sectors, especially for diseases that are underserved due to the high costs and extended timeline associated with de novo drug development. With the assistance of AI, drug repurposing has witnessed significant breakthroughs in the fight against global pandemics and AMR [105, 106]. A total of 4707 compounds, including 3422 marketed drugs, were collected in an online Drug Repurposing Hub [107]. Two steps were employed to identify drugs with repurposing potential. First, all the existing drugs were collected and integrated into a database. Second, all the chemicals that had reached clinical development were selected and subjected to structure-matching analysis. After clustering analysis, the compounds were clustered into different groups using a self-organizing map algorithm. Researchers from the Massachusetts Institute of Technology used ML-based AI to identify novel antibacterial molecules from the Drug Repurposing Hub [108]. For example, halicin, an anti-diabetic drug, has bactericidal effect by damaging the electrochemical gradient of the bacterial membrane [108]. From this conserved MOA, halicin was shown to display antimicrobial activity against a broad spectrum of pathogens, such as *M*. *tuberculosis*, carbapenem-resistant *Enterobacteriaceae*, *Clostridium difficile*, and *A. baumannii* [109]. Besides, the naive Bayesian approach combined with whole-cell screening identified 5 molecules from the GlaxoSmithKline antimalarial database with potent activity against *M. tuberculosis* activity, showing repurposing potential [110]. Furthermore, support vector machines and random forest methods were combined to construct an antibacterial compound predictor using chemicals with known antibacterial properties from the ChEMBL database [111]. The resulting predictor was then employed to screen FDA-approved small molecules from the DrugBank database, identifying 1087 compounds predicted to have potential antibacterial activities. Notably, among the 1087 compounds, 154 are already FDA-approved antibacterial drugs. Encouraged by this result, 8 compounds with novel structures were selected for further experimental validation [111]. These innovative approaches provide new insights for antibacterial discovery and will undoubtedly contribute to new antimicrobial development.

### AI in AMP discovery

AMPs are a class of structurally diverse small peptides based on their antimicrobial activities, typically containing a few to dozens of amino acid residues in their sequence, with or without further modifications, exerting their antimicrobial activities through a variety of mechanisms [112]. AMPs are proposed to target cell membranes, demonstrating the capability to bind and disrupt both negatively charged and zwitterionic membranes, ultimately leading to membrane permeabilization. This specific MOA positions AMPs as a new class of potential antibacterial drugs, which likely makes it challenging to develop resistance [46, 112].

#### AMP mining from extant sequence space

**Fig. 3:**
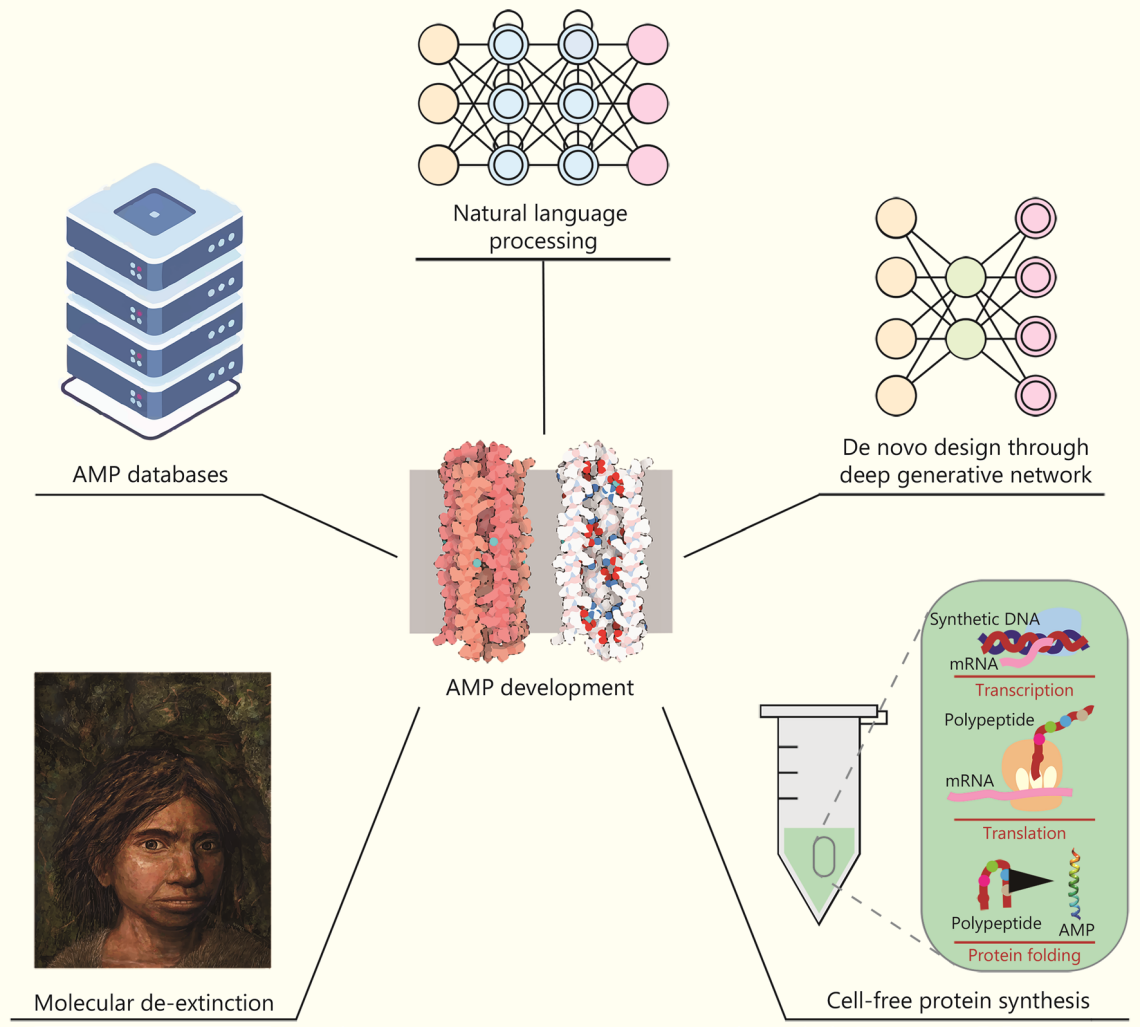
Artificial intelligence (AI) in the development of antimicrobial peptides (AMPs). AMPs databases have laid a solid foundation for AI-based model training, including natural language processing and deep generative networks. AI models can then be used to mine a wide range of protein sequence space, including the extinct human proteome, while high-throughput methods like cell-free synthesis significantly accelerate the speed of validation of candidate AMPs

Novel AMP development has also benefitted substantially from the involvement of AI [113] (Fig. 3). Platforms such as Deep-AmPEP30 (a DL-based platform) [114], IAMPE (a webserver with ML algorithms) [115], and DeepACP (a deep recurrent neural network-based model) [116] have greatly facilitated novel peptide discovery and synthesis. Besides, there are well-maintained databases of AMPs derived from genetic sequences [117]. For instance, the AMPer database aims to classify natural and novel AMPs (including 1045 mature peptides and 253 peptides) by using hidden Markov models [118]. ANTIMIC collects approximately 1700 AMPs from eukaryotes and prokaryotes, which serve as templates for designing novel AMPs [119]. In addition, synthetic peptide design has helped to unravel the underlying SARs. There are also advanced strategies employed for potential AMPs design. Firstly, AMPs can be modified based on known AMPs, aiming to generate peptides with greater antimicrobial activity and/or reduced toxicity [120]. Efforts to design and synthesize protein epitope mimetics (PEMs) often lead to novel AMPs. Various PEMs, like β-hairpin structures and above mentioned LptD binding peptide murepavadin, are designed based on PEMs [121]. Biophysically motivated modeling studies have also been applied to understand AMP activity and design AMPs. Examples of modeling strategies include Gibbs free energy perturbation, molecular dynamics simulations, as well as thermodynamics calculations of the interactions between AMPs and cell membranes [122]. Bactenecin [122] and an indolicidin analog CP-11 [123] are examples whose activities were studied and designed through this approach, respectively. In addition, Wu et al. [124] employed an amino acid-based activity prediction method that resulted in the identification of DP7. Compared to its parent peptide HH2, DP7 exhibits improved activity against *S. aureus* and lower cytotoxicity.

NLP is a type of DL that applies computer technology to the analysis and synthesis of natural language and speech [125]. Ma et al. [126] created a prediction method for AMPs by integrating 3 NLP neural network models—Long short-term memory, Attention, and Bidirectional Encoder Representations from Transformer. After learning from thousands of existing AMP sequences, they achieved a precision rate exceeding 90%, which is superior to previous models based on amino acid composition and properties for determining AMPs. Based on 2349 peptide sequences identified in human gut microbiome data as candidate AMPs, 216 synthetic AMPs were designed, and 181 were confirmed to have antimicrobial activity. Finally, 11 of these AMPs showed highly potent efficacy against multidrug-resistant Gram-negative bacteria [126]. This study showed the great potential of combining ML and large meta datasets, such as omics datasets, to improve the efficiency of AMP prediction and identify AMP molecules with new MOAs. By combining microbiome big data and the latest DL models, this study sets an excellent example of AI + microbiome molecular mining and transformation, showcasing the high feasibility of using computational methods to discover active molecules (such as proteins or RNA) for therapy from other types of omics data.

#### AMP mining from extinct and virtual sequence space

Microbes are not the sole source for AMP mining. Based on the concept of “molecular de-extinction”, Maasch et al. [127] developed panCleave, a random forest model capable of predicting proteome-wide cleavage sites to prospect for AMPs encrypted within extinct and extant human proteomes. Lead AMPs identified by panCleave exhibited membrane permeabilization and anti-infective efficacy against *A. baumannii* in both murine skin abscess and thigh infection models, highlighting the potential of paleoproteome as a reservoir for drug candidates [127]. AMP mining from existing proteomes, regardless of the source, is inevitably biased towards the sequence space confined by the proteome. To explore the entire space of peptide sequences, Huang et al. [128] developed a sequential model ensemble pipeline comprised of ML modules with a coarse-to-fine design principle to mine the entire virtual library of peptides with length of six-to-nine amino acids. Three lead hexapeptides identified by this pipeline exhibit potent activity against multidrug-resistant clinical isolates in both in vitro and in vivo models, indicating the great potential of sequential model ensemble pipeline for unbiased peptide screening tasks [128].

#### De novo design of AMP

AMPs have also been designed de novo through deep generative neural networks [129]. Researchers from International Business Machines Corporation employed two types of variational inference autoencoder [129]: the classic Variational Autoencoder (VAE) and the Wasserstein Autoencoder, to design two novel and highly active AMPs [130]. In this study, the peptide generation problem is represented as a density modeling mathematical problem; the model samples the peptide sequence space in a way that primarily involves the regions with high probability density. The density estimation algorithm has been adjusted to assign high likelihoods to known peptides and “punish” random meaningless sequences [130]. Additionally, the researchers used 1.7 million peptide sequences from the UniProt database to train the algorithm. The contact frequency between positive residues and lipid bilayers was used as a predictive metric for antimicrobial activity [130]. After high-throughput in silico screening, only 20 peptide sequences were retained. They were transferred to wet laboratories for characterization of their antimicrobial activities against model bacterial strains, including Gram-positive *S*. *aureus* and Gram-negative *E*. *coli*. Within just 48 d, two novel AMPs with broad-spectrum antimicrobial activity were discovered [130]. Another endeavor to design AMPs using a deep generative model was achieved by Szymczak et al. [131] who proposed HydrAMP, a conditional VAE that performs both analog generation and unconstrained generation. The AMPs showed potent activities against 5 bacterial strains (both Gram-positive and Gram-negative) including those that are antibiotic-resistant. This demonstrates that HydrAMP represents a remarkable advancement in designing novel AMPs with high potency to combat antibiotic resistance [131].

One consequence of the de novo design of AMPs through deep generative neural networks is the large cohort of candidate peptides for in vitro validation. Compared to chemical synthesis, DNA-based bioproduction significantly increases the throughput for peptide screening, but cell-based methods are limited due to the toxicity of peptides to their bacterial chassis [132]. To address this limitation, Pandi et al. [132] developed a cell-free protein synthesis pipeline to test 500 DL-based de novo designed AMPs, from which 6 AMPs displayed broad-spectrum activity against multidrug-resistant bacterial isolates, indicating the power of DL-based design and cell-free protein synthesis in AMP development.

### AI in phage therapy development

**Fig. 4:**
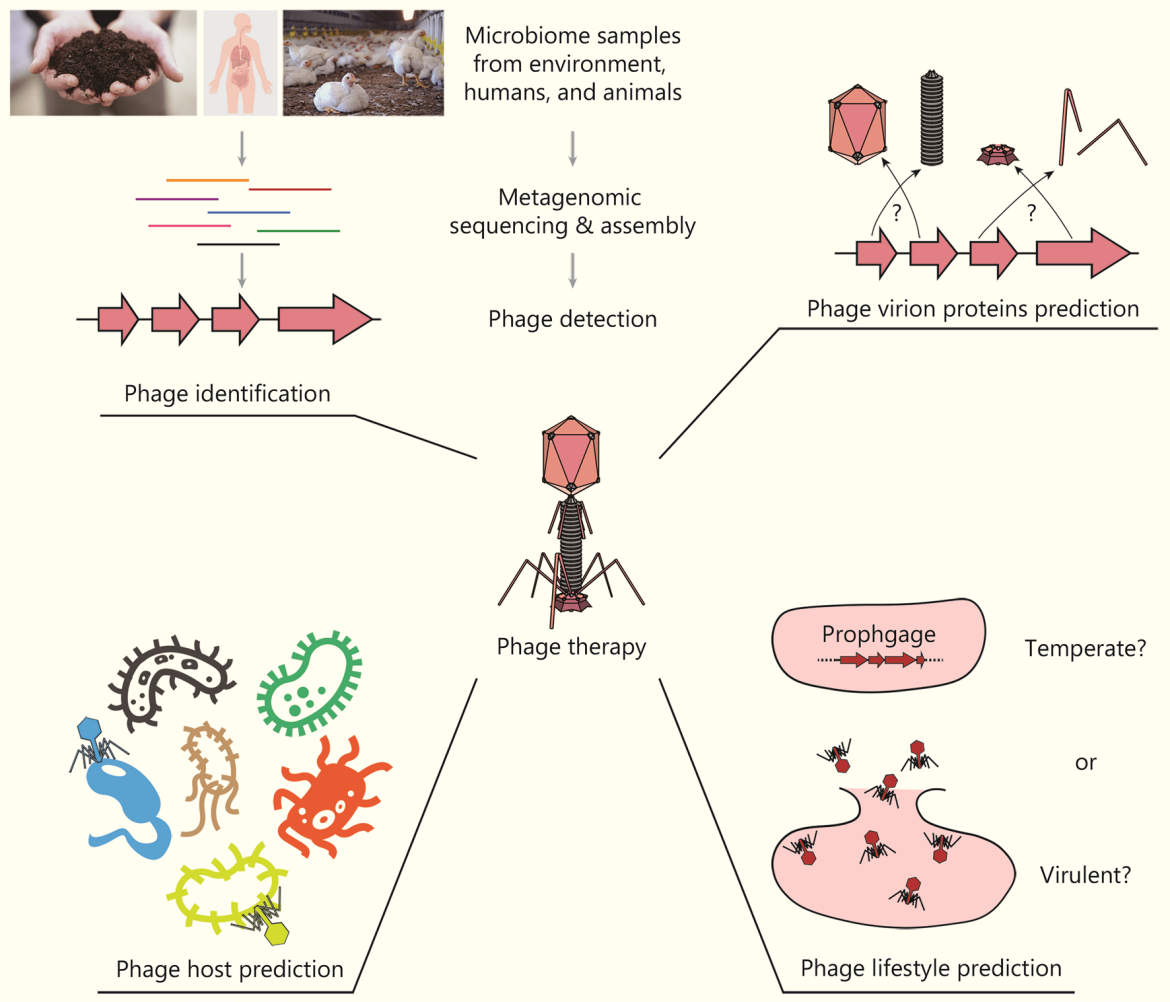
Artificial intelligence (AI) in the development of phage therapies. AI-based models have played a significant role in studying phages from their natural sources. This includes identifying phages from metagenomic samples, annotating phage virion proteins from phage genome sequences, predicting phage hosts, and determining phage lifestyles. These efforts lay a solid foundation for developing novel phage therapies

In addition to small-molecule agents and AMPs, alternative strategies have also played vital roles in the battle against antibiotic resistance, one of them being phage therapy [133, 134]. Bacteriophages, natural predators of bacteria, have co-evolved with their bacterial hosts for 3.8 billion years and form an integral part of the human microbiome [135]. Compared to antibiotics, which are mostly broad-spectrum, phage therapy offers remarkably higher specificity, minimizing disturbances to the microbiota and preventing antibiotic-induced AMR dissemination [136, 137]. There have already been several examples of clinical success [138–141]. This section delineates the rational design of phage therapy into 4 consecutive steps: phage identification, prediction of phage virion proteins (PVPs), analysis of phage lifestyle, and exploration of phage-host interactions, providing a review of the contribution of AI in each stage (Fig. 4).

#### Phage identification

Recent advances in metagenomic sequencing have highlighted the pivotal role of viruses in various ecosystems [142]. To address this growing significance, innovative AI-driven tools have been developed for the discovery, annotation, and analysis of viral sequences within complex metagenomic datasets [143, 144]. Seeker, for instance, is a DL-based tool that swiftly detects diverse bacteriophages, even in cases where they exhibit minimal sequence similarity to known phage families [145]. Meanwhile, VIBRANT employs a hybrid ML and protein similarity approach to autonomously recover, annotate, and assess the metabolic impacts of viruses in metagenomic assemblies, surpassing traditional virus identification programs [146]. In addition, VirSorter2 significantly enhances the accuracy and breadth of virus sequence detection in metagenomic datasets, utilizing multiple classifiers to detect a wide range of viruses with high precision [147]. Moreover, PhageBoost, a novel ML method based on feature space designed for fast and generalized prophage discovery, significantly enhances bacteriophage identification [148]. DEPhT is a multimodal tool tailored for the discovery, precise extraction, and annotation of prophages in *Mycobacterium* genomes, enabling detailed comparative genomic analyses through efficient discrimination between phage and bacterial sequences [149]. Furthermore, Phanta excels in virome-inclusive gut microbiome profiling, relying on *k*-mer-based classification methods and comprehensive gut viral genome catalogs to quickly and accurately quantify both prokaryotes and viruses, significantly improving viral species identification compared to assembly-based methods [150].

#### PVPs prediction

PVPs, encompassing capsid proteins, tail proteins, and phage particle enzymes, play a critical role in governing bacteriophage and interactions with their bacterial hosts [143]. Predicting PVPs is crucial as it enables the functional annotation of these proteins, improving our understanding of their potential utility in phage therapy, such as shelf-life, particularly valuable when a significant percentage of these proteins lack assigned functions due to low sequence conservation and limited experimental data.

To address this demand, multiple AI-driven initiatives have emerged. In an early pioneering effort, Seguritan et al. [151] developed ANNs to predict phage structural protein sequences, enabling the identification of virion structures even in the absence of significant similarities to known sequences. Subsequently, phage artificial neural networks (PhANNs) were introduced, employing an ensemble of ANNs to categorize phage open reading frames into 10 major structural protein classes or “other” category, delivering swift and precise functional annotations for diverse phage sequences [152]. In addition, STEP^3^ is a computational tool that harnesses evolutionary features in diverse phage genomes to achieve robust and accurate predictions, addressing the challenge of poor genome annotation and improving the quality of phage cocktails for more effective phage therapy [153]. Moreover, SCORPION employs a two-step feature selection strategy to construct a feature vector and build a stacked model, offering a cost-effective and scalable tool for the accurate identification of PVPs solely based on protein primary sequences [154].

#### Phage host prediction

The identification of host organisms for phages is of paramount importance in understanding viral-host relationships and advancing therapeutic applications. To address this challenge, innovative computational methods have been developed. VirHostMatcher-Net employs a flexible network-based framework, integrating CRISPR sequences and alignment-free similarity measures, which significantly improves host prediction accuracy and greatly expands the spectrum of known virus-host interactions [155]. Meanwhile, the development of a computational model based on genome analysis enables efficient prediction of phage-bacterium interactions, achieving a predictive performance of around 90% [156]. Furthermore, HostG, a semi-supervised learning model, leverages a knowledge graph and graph convolutional network to enhance host prediction for novel viruses, offering the valuable ability to predict hosts from new taxa [157]. These approaches collectively contribute to our ability to decipher viral-host relationships and explore the potential applications of bacteriophages in various domains.

#### Phage lifestyle prediction

Bacteriophages exhibit two contrasting lifestyles: virulent and temperate, with the virulent lifestyle bearing significant implications for phage therapy. The prediction of phage lifestyles is crucial but remains challenging, especially for phages constructed from metagenomic data. To address this issue, innovative AI-driven approaches have emerged. PHACTS (Phage Classification Tool Set) introduces a novel similarity algorithm and a supervised random forest classifier to accurately classify phages into virulent or temperate categories, achieving an impressive precision rate of 99% [158]. In parallel, BACPHLIP (BACterioPHage LIfestyle Predictor) employs conserved protein domains and a random forest classifier to predict the lifestyle of bacteriophages, attaining an outstanding accuracy of 98% [159]. Moreover, by harnessing Bidirectional Encoder Representations from Transformer, a contextualized embedding model inspired by NLP, to represent phage contigs, PhaTYP (Phage TYPe prediction tool) achieves outstanding performance in accurately predicting the lifestyles of phages from short contigs [160].

### AI in the discovery of antibacterial EOs

Another important class of non-traditional antibacterial agents is EOs. The antibacterial mechanism of EOs is complex, and various mechanisms have been proposed. The most common mechanism is that they penetrate through the cell wall and membrane, ultimately disrupting cell membrane integrity and causing cell death [161]. It has been also proposed that EOs can destabilize cellular architecture, affect the external envelope of the cell, and reduce membrane potential [162]. Predicting EOs’ bioactivities is challenging due to prevalent synergisms and antagonisms between EOs’ complex components. Thus, time-consuming disk diffusion assays are required for each new combination. ANNs were trained to predict the antimicrobial activity of EOs and extract fractions against 4 main pathogens (*S. aureus*, *E. coli*, *Candida albicans* and *Clostridium perfringens*) [163]. Notably, the ANN reached > 70% prediction accuracy within a 10 mm maximum error range, making it a reliable and fast tool for predicting the antimicrobial activities of EOs [163].

### AI in predicting MOA and resistance mechanisms of novel antibacterials

#### Public databases for resistance development prediction

**Fig. 5:**
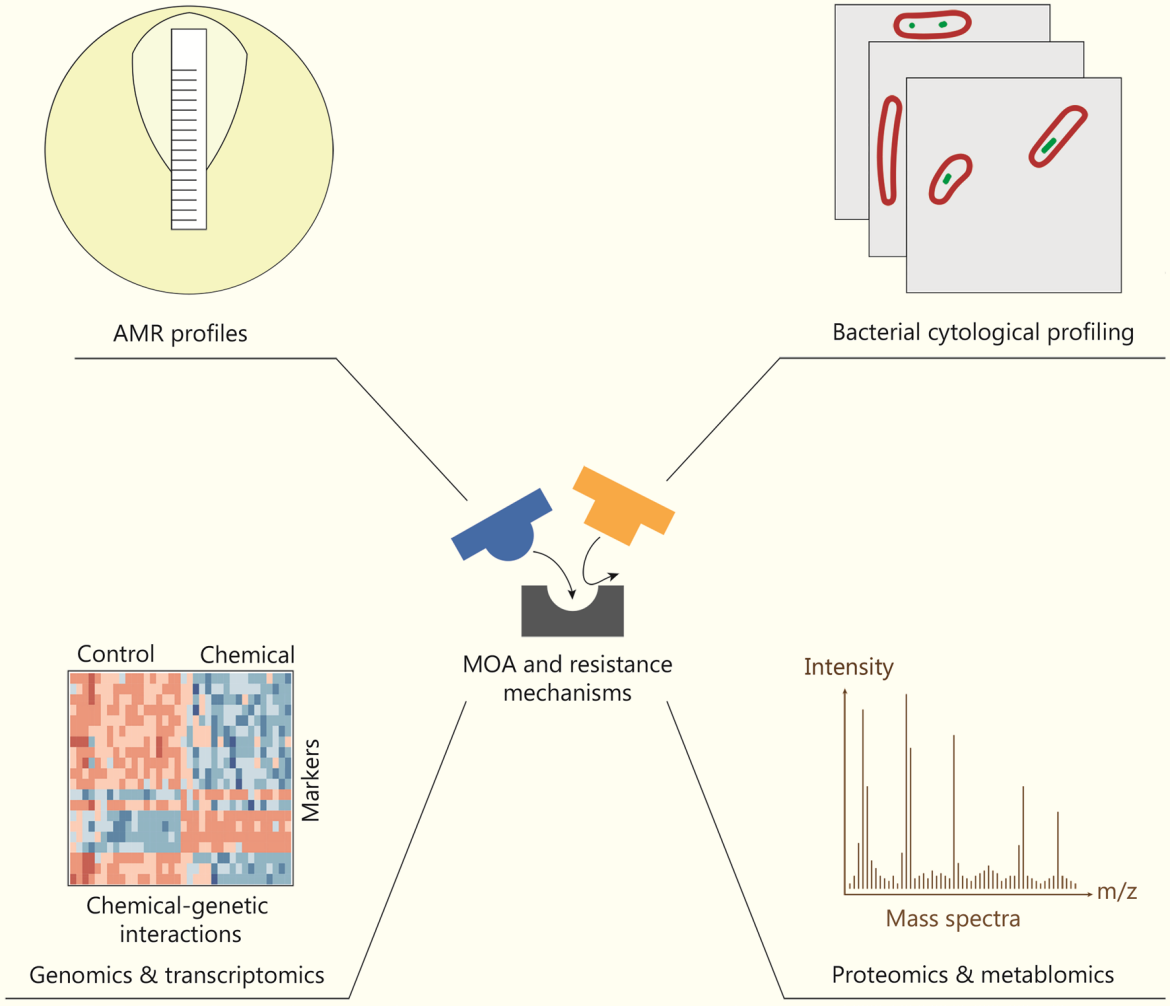
Artificial intelligence (AI) in deciphering the mechanisms of action (MOA) and resistance mechanisms of novel antibacterials. Comparing cellular responses of bacteria before and after treatment of an antibacterial compound through multidimensional profiling enables AI-based methods to delineate the MOA of compounds and predict mechanisms of arising resistance. AMR antimicrobial resistance

Resistance development is an inevitable consequence of any antibacterials and its mechanism is strongly linked to the MOA of the cognate drug. Therefore, it is essential to track, monitor and predict bacterial genome evolution, mutation developments and resistant genes epidemiology. ML-based approaches have been applied to mine bacterial genomic data to decipher resistance mechanisms and predict resistance development (Fig. 5). This is crucial for decision-making when developing drug candidates in advance of resistance development. BacMet (http://bacmet.biomedicine.gu.se) is a bacterial resistance gene database that has collected 753 bacterial resistance genes confirmed by experiments, and it contains 155,512 potential predicted resistance genes based on sequence similarity of the experimental-confirmed genes [164]. The Comprehensive Antibiotic Resistance Database (https://card.mcmaster.ca/) is a curated bioinformatic database of resistance genes, their products, and associated phenotypes. It includes 6627 ontology terms, 5010 reference sequences, 1933 single nucleotide polymorphisms, 3004 publications, and 5057 AMR detection models [165]. Comprehensive Antibiotic Resistance Database also provides predicted resistomes for 377 important pathogens including 21,079 chromosomes, 2662 genomic islands, 41,828 plasmids, 155,606 whole-genome sequencing assemblies, and 322,710 alleles (data released in July 2022). The Bacterial Diversity Metadatabase (https://bacdive.dsmz.de/about) is currently the largest database with standardized bacterial phenotypic information, containing 81,827 bacterial and archaeal strains, including 14,091 strains which covers approximately 90% of species [166]. Furthermore, libraries such as Plasmid ATLAS [167], and Virulence Factor Database [168] provide solid support for predictions against plasmid-borne genetic factors and virulence factors, respectively.

#### AI in phenotype-assisted prediction of resistant determinants

Publicly available databases alone do not provide a complete solution; traditional approaches relying on searching for known resistant determinants in existing databases are intrinsically biased towards known antibiotic-resistance genes (ARGs), resulting in high false negative rates [169]. To overcome such limitations, bacterial genome sequences and antimicrobial susceptibility testing (AST) metadata have been exploited to train various ML-models [170], enabling downstream prediction tasks [171]. For instance, PATRIC (http://patricbrc.org/) is a database capable of predicting phenotypes and identifying of genomic regions relating to resistance [172]. Building on PATRIC, AdaBoost ML classifiers were built to identify carbapenem resistance in *A. baumannii*, methicillin-resistant *S. aureus*, and β-lactam resistance in *K. pneumoniae*, utilizing whole-genome bacterial data and MICs [173]. Later, Moradigaravand et al. [169] demonstrated that the gradient boosted decisions tree model stood out as the best ML model for predictions the resistance of *E. coli* against eleven compounds across 4 major antibiotic classes. Moreover, Her et al. [174] took a pan-genome-based approach and found that AMR genes are unevenly distributed in the core and accessory genomes. Dividing the pan-genome into core and accessory parts further improved prediction accuracy in *E. coli*.

Large and high-quality training sets enable ML models to reach even higher predictive power. Nguyen et al. [175] also employed an extreme gradient boosting (XGboost)-based ML model, achieving prediction not only for resistance but also for MIC in nontyphoidal *Salmonella*. Furthermore, the integration of transcriptomes and genomes could further enhance the predictive powers of ML-based classifiers to predict AMR phenotypes in Gram-negative ESKAPE pathogens. A pioneering work by Bhattacharyya et al. [176] exploited differences in early transcriptional changes between sensitive and resistant strains upon antibiotic treatments to train an ML classifier, resulting in GoPhAST-R, a rapid assay with high classifying accuracy on *E. coli*, *K. pneumoniae*, and *A. baumannii*. Following this, Khaledi et al. [177] took a similar approach by integrating single nucleotide polymorphisms, gene presence/absence and transcriptomic profiles measured in unstressed conditions, obtaining an ML model with high predictive value and sensitivity on *P. aeruginosa*.

In addition to predicting the resistance phenotype of individual strains, ML-based approaches have also been used to interrogate metagenomic data. DeepARG, a DL-based model built on a dissimilarity matrix created from all known categories of ARGs, demonstrates both high precision and recall [178]. DeepARG has two versions: SS and LS, tailored to handle short and long sequence reads from metagenomic libraries, respectively [178]. Moreover, using an ML-based approach, it was found that genes such as subclass β-lactamases and vancomycin resistance regulators play a crucial role in determining the bacterial survival upon antibiotic treatment in premature infant gut microbiome [179].

Interpretation of ML models could go beyond resistance prediction and even offer mechanistic insights. An ML model trained using genome sequences of *K. pneumoniae* exhibiting polymyxin resistance (PR) identified not only known PR genes but also several stress response genes as potential novel PR determinants [180]. This theme was recapitulated by Sunuwar et al. [181] who invented a bioinformatics framework to predict loci underlying AMR phenotypes.

#### AI in functional annotation of antibacterials through integrated profiling

Adaptation of bacteria to antibiotics is a complex process, often involving systematic alterations of cellular pathways at multiple levels. Therefore, in addition to the traditional view where drug-target interactions are the major focus, a more holistic perspective is required to provide deeper insights into bacterial resistance development and evolution. Fortunately, the advances in molecular measurement technologies enable multi-dimensional profiling of features of bacteria upon drug treatments, including both macroscopic cell morphology and microscopic omics (genome, transcriptome, proteome and metabolome) characteristics [182]. Moreover, when such profiling is integrated with compound library screening and genome editing technologies, it has enabled the coupling of physicochemical properties of compounds with their phenotypic and molecular downstream impacts, revealing MOAs and resistant mechanisms of compound libraries [182].

The high dimensional and heterogeneous nature of drug profiling data types inevitably brings new challenges in data processing and interpretation, making ML algorithms particularly efficient tools for data mining [183]. In the following section, specific examples of coupling of drug multi-dimensional profiling and ML classifier models from multiple levels will be discussed.

At the level of cell morphology, bacterial cytological profiling is a fluorescence microscopy-based method capable of identifying cellular targets of antibacterial compounds with high distinguishing power and throughput [184]. The development of DL-based image processing algorithms, such as resolution enhancement [185], has led to the creation of platforms like MorphEUS, designed to classify drugs against *M. tuberculosis* [186]. In some cases, bacterial cytological profiling fails to classify a compound in any existing MOA class, such as in the case of SCH-79797; however, it often suggests a novel MOA [187]. Further characterization combining genetic, proteomic, metabolomic, and cell-based assays, demonstrated that SCH-79797 is a dual-mechanism drug targeting both folate metabolism and membrane integrity [187].

At the transcriptome level, by integrating the growth responses to different drug combinations, transcriptome profiling to single drugs, and transcription factor regulatory networks of *M. tuberculosis*, Ma et al. [188] developed INDIGO-MTB, an ML-based classifier capable of predicting drug synergy for treating *M. tuberculosis* infections. Additionally, it identified Rv1353c as a transcription factor regulating multiple drug interactions.

At the level of proteome, Weis et al. [189] combined ML algorithms and MALDI-TOF mass spectrometry to yield substantial acceleration of AMR determination. They collected mass spectrometry data from clinical strains using Bruker’s MALDI Biotyper microbial mass spectrometry system and coupled this data with strains’ resistance profiles to create the DRIAMS dataset [189]. The dataset includes 768,300 pieces of resistance data for more than 70 antimicrobials and MALDI-TOF mass spectrometry data for more than 300,000 clinical strains of 803 pathogens. They used datasets to train three ML algorithms: logistic regression, a deep neural network classifier (multilayer perceptron, MLP), and gradient-boosted decision trees (LightGBM). Applying AUROC as the performance metric, MLP and LightGBM were demonstrated to be the best-performing classifiers [189]. Therefore, this new approach is anticipated to be a dependable and efficient way to improve antibiotic stewardship and optimize infection treatment regimens in the future.

At the level of metabolome, using the metabolic responses of *Mycobacterium smegmatis* to 62 reference compounds with known MOAs, Zampieri et al. [190] established a drug-metabolome profile compendium that was used to predict the MOAs of 212 novel antimycobacterial compounds. This approach managed to predict the MOAs of 77% of novel compounds, some of which were experimentally validated [190]. To further explore phenotypic space potentially unreachable by existing reference compounds, Anglada-Girotto et al. [191] exploited CRISPR interference (CRISPRi) technology to systemically knockdown 352 essential genes in *E. coli* and combined it with non-targeted metabolomics to generate a reference map of metabolic changes. When compared with 1342 drug-induced metabolic changes, this integrated approach succeeded in high-throughput de novo functional annotations of novel antibacterials [191].

In contrast to conventional ML techniques, which explore and extract information from large omics or systematic datasets to understand relationships between antibiotic treatments and cellular phenotypes but create “black-boxes” where only correlative relationships are identified, the “white-boxes” approach, based on genome-scale metabolic network model (GSMM), was reported to establish causal relationships that aid mechanistic interpretation [192]. GSMM integrates both experimental (incorporating transcriptomic, proteomic, and biochemical screening) and computational modeling (network modeling and ML) to form an ML approach to reveal the MOAs of antibiotics [192]. Through these models, complicated cellular responses are integrated, and key metabolic pathways are extracted by constraint-based methods. Yang et al. [192] constructed a GSMM and demonstrated that purine biosynthesis pathway, central carbon metabolism, and redox status are crucial pathways for bacterial survival to antibiotics. CarveMe is an open-source, user-friendly automated tool designed to construct GSMM of bacterial species. It employs a top-down approach that begins with building a universal single-species model and then incorporates manually curated models to reproduce experimental phenotypes [193]. Currently, CarveMe has created a database of 5587 bacterial models for users to apply and understand the key cellular responses to antibiotics.

### AI in antibiotic stewardship

Antibiotic misuse is a strong driving force for the emergence and exacerbation of AMR. Accurate prescriptions should be based on the AST results of the infecting pathogen, but standard AST takes days. Therefore, in practical terms, the demand for rapid clinical intervention often necessitates empirical drug prescriptions in the absence of AST data, posing the risk of ineffective treatment and AMR development [194]. Advanced molecular profiling methods, such as those discussed in Sect. 2.5, inevitably require powerful equipment and technical expertise and hence are still at the laboratory stage. Therefore, the clinical demand for a rapid, accessible way of predicting bacterial antimicrobial susceptibilities to guide prescription is significant, and AI has again been deeply involved to meet it [195].

By examining a 10-year longitudinal data set encompassing 711,099 community-acquired UTIs from 315,047 patients, Yelin et al. [196] found strong correlations between antibiotic resistance and parameters obtained from the clinical history of patients. The integration of those correlations gave rise to ML algorithms capable of predicting drug-specific antibiotic resistance in a personalized fashion, and a 1-year retrospective testing demonstrated that the ML algorithms significantly reduced the rate of mismatched treatment from over 8% to below 6% [196]. In addition, Kanjilal et al. [197] developed an ML decision algorithm using electronic health record data from over 10,000 patients with uncomplicated UTIs from 2007–2013 in Boston to recommend the narrowest possible antibiotic. When evaluated on a test cohort of 3629 patients retrospectively, compared to clinicians, this algorithm achieved a 67% reduction in the use of second-line antibiotics [197]. Similar endeavors to exploit ML to personalize antibiotic selection were done in the UK and Greece [198, 199].

ML goes beyond merely reducing the rate of mismatched treatments; it extends its capabilities to prevent post-treatment resistance-gaining bacterial recurrences [200]. Combining whole-genome sequencing and ML analysis of a large-scale longitudinal database of urinary and wound clinical isolates pre- and post-treatment from the same patient, Stracy et al. [201] found that treatment-induced resistance emergence is largely caused by re-infection of a different resistant clone rather than de novo resistance evolution. Moreover, as a recurrence-causing resistant clone most frequently comes from a patient’s microbiota [202], it is in many cases reflected by the patient’s infection history, which could be analyzed by ML algorithms to offer personalized treatment recommendations to reduce the risks of treatment-induced AMR [201].

## Conclusions and future perspectives

In conclusion, despite AMR posing a serious threat to human health and having garnered extensive attention, the development of antimicrobials has been slow. Only 18 novel antibiotics were approved from 2014 to 2021, with only 2 possessing a novel MOA. By analyzing antibiotics currently in clinical development, it has been revealed that novel antibiotic development is showing two trends: 1) from synthetic small molecules to biologicals; and 2) from broad spectrum to narrow spectrum. To meet ever-pressing clinical demands, AI has already been involved in the discovery of novel AMPs and EOs, drug repurposing, and resistance mechanism prediction (Table 2).

**Table 2:** Antimicrobial drugs whose development has utilized computational and artificial intelligence (AI) technologies

Antibacterial drugs	Development stage	Technology	Targeted pathogens
SPR206	Completed Phase I	SAR-based design	CRAB, CRPA, CRKP
QPX 9003	Phase I	SAR combining with STR and SPR-based design	CRAB, CRPA, CRKP
MRX-8	Phase I	Soft drug design	CRAB, CRPA, CRKP
IB-367 (iseganan)	Failed in Phase III	Molecular dynamics simulation	Gram-negative and Gram-positive pathogens and *Candida albicans*
Reltecimod (AB103)	Phase III	Molecular dynamics simulation, binds to the CD28 receptor	Modulate the host’s immune response in severe Gram-negative bacterial infections
Murepavadin	Terminated Phase III	Protein epitope mimetics	CRPA
Bactenecin	Preclinical	Machine-learning classifier	*P. aeruginosa*
Indolicidin analogue CP-11	Preclinical	Biophysically motivated modeling	Gram-negative and Gram-positive pathogens, fungi, and viruses
Halicin	Preclinical	Drug repurposing Hub	*M. tuberculosis*, *C. difficile*, *A. baumannii*, and CRE

*CRAB* carbapenem-resistant *A. baumannii*, *CRPA* carbapenem-resistant *P. aeruginosa*, *CRKP* carbapenem-resistant *Klebsiella pneumoniae*, *CRE* carbapenem-resistant *Enterobacteriaceae*, *SAR* structure–activity relationships, *STR* structure–toxicity relationship, *SPR* structure-pharmacokinetic relationship, *P. aeruginosa Pseudomonas aeruginosa*, *M. tuberculosis Mycobacterium tuberculosis*, *C. difficile Clostridioides difficile*, *A. baumannii Acinetobacter baumannii*

In the future, AI is likely to be involved in more stages of drug development, including molecular design, prediction of dosage regimens and associated effectiveness, dynamic modeling of protein–protein interactions [203], ligand-based simulation, quantitative structure–activity relationship modeling, molecular representations and high-order feature extraction [204], drug repurposing, and reduction of toxicity. With the substantial advantages of AI, potent chemical entities can be investigated at an unprecedentedly rapid pace compared to traditional methods, ultimately reducing drug development costs for research and development. This has the promise of increasing the success rate of clinical trials. The rapid advances of AI, combined with the current big data era (large datasets from high-throughput technologies, genomic/expression databases), should accelerate the emergence of new chemical entities possessing novel MOAs. As a result, more antibiotics or antibacterial biologicals are expected to make it through the drug development pipeline and enter the market. With the hope of combining new drugs and antibiotic stewardship, the fight against resistant bacteria should significantly benefit from the slower development of antibiotic resistance.

Furthermore, despite their high predictive performance, most current AI systems, particularly those that rely on deep neural networks, still suffer from several limitations. Firstly, DL can be prone to failures due to shortcut learning, namely decision rules that excel in standard benchmarks but struggle to adapt when tested under more demanding conditions [205]. This phenomenon often occurs in the later stages of tasks like drug design, which limits their use in decision-making [206]. The likelihood of failure can be reduced by calculating forecast uncertainty to prevent making poor decisions. Moreover, the model’s decision-making can be examined and its reliability increased by expert supervision [207]. Secondly, a large proportion of existing AI models remain “black-boxes” models with low interpretability, meaning that humans cannot comprehend the rules behind decisions made [208]. This lack of transparency and accountability can lead to severe consequences, especially when it comes to high-stake decisions such as drug recommendations [208]. Therefore, despite the great challenges ahead, the direction toward more explainable AI is inevitable [209]. Thirdly, data availability is the key. AI has significantly shortened the in vitro stages in the drug-discovery pipelines of novel antimicrobials. However, little aid has been gained from AI regarding how those drugs behave in the human body, especially for biologicals due to their complex nature. The available data required to train AI models to address questions in vivo is far from sufficient [210]. To address this issue, it is crucial to strike a balance between data accessibility and confidentiality. Several promising avenues worth further exploration include federated learning, active learning, and swarm learning [210, 211].

For any AI-based antimicrobial research, one can never overstate the importance of high-quality wet experimental data [207]. A future challenge will be the integration of drug discovery with disease progression, biomarker discovery, and precision medicine. With the advances in high-throughput and single-cell technologies [212, 213], AI could help build systematic models to rapidly diagnose diseases and automatically recommend more personalized treatments for patients. It could also encourage pharmaceutical companies to transition from biomarker discovery to drug development, revolutionizing future drug discovery and clinical practice [214].

AMR, due to the inappropriate overuse of antimicrobials, represents a complex challenge that extends beyond the development of new drugs and therapeutics. Resolving this problem, also requires a collaborative and integrated educational approach to the proper use of antibiotics by healthcare professionals and the public.

## Data Availability

Not applicable.

## Abbreviations

*A*. *baumannii*: *Acinetobacter baumannii*
AI: Artificial intelligence
AMPs: Antimicrobial peptides
AMR: Antimicrobial resistance
ANNs: Artificial neural networks
BGCs: Biosynthetic gene clusters
DL: Deep learning
*E. coli*: *Escherichia coli*
EOs: Essential oils
FDA: Food and Drug Administration
GSMM: Genome-scale metabolic network model
*K*. *pneumoniae*: *Klebsiella pneumoniae*
LptD: Lipopolysaccharide transport protein D
*M. tuberculosis*: *Mycobacterium tuberculosis*
ML: Machine learning
MOA: Mechanisms of action
NLP: Natural language processing
*P*. *aeruginosa*: *Pseudomonas aeruginosa*
PEMs: Protein epitope mimetics
PVPs: Phage virion proteins
PR: Polymyxin resistance
*S*. *aureus*: *Staphylococcus aureus*
SAR: Structure–activity relationships
SPR: Structure-pharmacokinetic relationship
STR: Structure–toxicity relationship
UTIs: Urinary tract infections
WHO: World Health Organization
